# A Digital Compendium of Genes Mediating the Reversible Phosphorylation of Proteins in Fe-Deficient *Arabidopsis* Roots

**DOI:** 10.3389/fpls.2013.00173

**Published:** 2013-06-03

**Authors:** Ping Lan, Wenfeng Li, Wolfgang Schmidt

**Affiliations:** ^1^State Key Laboratory of Soil and Sustainable Agriculture, Institute of Soil Science, Chinese Academy Sciences, Nanjing, China; ^2^Institute of Plant and Microbial Biology, Academia Sinica, Taipei, Taiwan

**Keywords:** protein phosphorylation, RNA-seq, co-expression, iron deficiency, potassium homeostasis

## Abstract

Post-translational modifications of proteins such as reversible phosphorylation provide an important but understudied regulatory network that controls important nodes in the adaptation of plants to environmental conditions. Iron (Fe) is an essential mineral nutrient for plants, but due to its low solubility often a limiting factor for optimal growth. To understand the role of protein phosphorylation in the regulation of cellular Fe homeostasis, we analyzed the expression of protein kinases (PKs) and phosphatases (PPs) in *Arabidopsis* roots by mining differentially expressed PK and PP genes. Transcriptome analysis using RNA-seq revealed that subsets of 203 PK and 39 PP genes were differentially expressed under Fe-deficient conditions. Functional modules of these PK and PP genes were further generated based on co-expression analysis using the MACCU toolbox on the basis of 300 publicly available root-related microarray data sets. Results revealed networks comprising 87 known or annotated PK and PP genes that could be subdivided into one large and several smaller highly co-expressed gene modules. The largest module was composed of 58 genes, most of which have been assigned to the leucine-rich repeat protein kinase superfamily and associated with the biological processes “hypotonic salinity response,” “potassium ion import,” and “cellular potassium ion homeostasis.” The comprehensive transcriptional information on PK and PP genes in iron-deficient roots provided here sets the stage for follow-up experiments and contributes to our understanding of the post-translational regulation of Fe deficiency and potassium ion homeostasis.

## Introduction

Iron (Fe) is an essential element for all living organisms. In plants, Fe is required for basic redox reactions in photosynthesis and respiration and for many vital enzymatic reactions such as DNA replication, lipid metabolism, and nitrogen fixation. Although Fe is one of the most abundant elements in the earth’s crust, its bioavailability is severely restricted due to an extremely low solubility at neutral or basic pH. Approximately 30% of the cultivated plants are grown on calcareous soils, making Fe deficiency a major constraint for crop yield and quality (Rellan-Alvarez et al., 2011). Excess Fe is cytotoxic due to the formation of potentially harmful reactive oxygen species. Thus, plants must tightly regulate Fe homeostasis to allow an effective acquisition, distribution, and utilization of Fe.

Plants have evolved sophisticated mechanisms to promote Fe availability. Fe is acquired by two distinct strategies, referred to as strategy I and strategy II (Romheld and Marschner, 1986). In strategy II plants such as maize (*Zea mays*), Fe(III) is chelated by phytosiderophores that are synthesized and secreted by plant roots and the Fe-phytosiderophore complex is taken up by an oligopeptide transporter, YELLOW-STRIPE1 (Curie et al., 2001). Strategy II is confined to the grasses. In strategy I plants such as *Arabidopsis* (*Arabidopsis thaliana*), Fe acquisition is controlled by two basic helix-loop-helix (bHLH) transcription factors, FER-LIKE IRON DEFICIENCY-INDUCED TRANSCRIPTION FACTOR (FIT) and POPEYE (PYE), regulating non-overlapping subsets of genes with various roles in Fe uptake and metabolism (Colangelo and Guerinot, 2004; Bauer et al., 2007; Long et al., 2010; Schmidt and Buckhout, 2011). Disruption of FIT or PYE function leads to severe growth reduction and chlorosis under Fe-limited conditions, implicating that the function of these genes is critical for regulating Fe homeostasis. FIT forms heterodimers with bHLH38 and bHLH39 and positively regulates a subset of Fe-responsive genes, including three key genes required for Fe acquisition that encode the ferric chelate reductase FERRIC REDUCTION OXIDASE2 (FRO2), the Fe transporter IRT1 (Eide et al., 1996; Robinson et al., 1999; Vert et al., 2002; Colangelo and Guerinot, 2004; Yuan et al., 2008), and the H^+^-translocating P-type ATPase AHA2 (Santi and Schmidt, 2009; Ivanov et al., 2012). Recent studies showed that the transcription factors bHLH100 and bHLH101, belonging to the Ib subgroup bHLH proteins, are also involved in *Arabidopsis* Fe-deficiency responses by interacting with FIT (Wang et al., 2013) or via a FIT-independent manner (Sivitz et al., 2012). *PYE* is preferentially expressed in the pericycle and aids in maintaining Fe homeostasis by positively regulating a separate set of genes. The expression of *BRUTUS* (*BTS*), encoding a putative E3 ligase protein that negatively regulates some of the Fe-deficiency responses, is tightly correlated with *PYE* gene activity. Both proteins interact with the PYE homologs IAA-LEU-RESISTANT3 (ILR3) and bHLH115, suggesting a complex and dynamic regulatory circuit that adapts plants to fluctuating availability of Fe (Long et al., 2010).

For long-distance transport, Fe is exported from the cell by the ferroportin ortholog IREG1/FPN1 and transported in the xylem as a complex with citrate (Morrissey et al., 2009). The MATE transporter FRD3 was shown to be important for the proper transport of Fe from roots to leaves. *frd3* mutants showed constitutive up-regulated Fe-deficiency responses, chlorotic leaves, and ectopic accumulation of Fe in the root vasculature (Rogers and Guerinot, 2002; Durrett et al., 2007). FRD3 loads citrate into the xylem, which is crucial for the transport of Fe to the shoot. A recent report further showed that FRD3 promotes Fe nutrition of symplastically disconnected tissues such as pollen throughout the development (Roschzttardtz et al., 2011).

The signaling processes that are upstream of or parallel to FIT, PYE, and BTS are largely unknown. All three genes are regulated by the plant’s Fe status, indicating that other components are involved in Fe sensing and signaling. The turnover of FIT is 26S proteasome-dependent (Lingam et al., 2011; Meiser et al., 2011; Sivitz et al., 2011), and the activity of IRT1 is post-translationally regulated by monoubiquitin (Barberon et al., 2011). Other post-translational processes, such as protein phosphorylation, were shown to be involved in the Fe-deficiency response (Lan et al., 2012b), but only for a few cases clear-cut evidence for a regulatory function of such modifications has been provided (Arnaud et al., 2006). An estimated one-third of all eukaryotic proteins are putatively regulated by reversible phosphorylation via protein kinases (PKs) and phosphatases (PPs), demonstrating the importance of this process. Phosphorylation can affect the configuration, activity, localization, interaction, and stability of proteins, thereby regulating crucial processes in metabolism and development. Transcriptional profiling experiments on Fe-deficient roots revealed several differentially expressed protein kinase genes, suggesting that alterations in protein phosphorylation patterns induced by Fe deficiency are involved in the control of Fe homeostasis (Colangelo and Guerinot, 2004; Dinneny et al., 2008; Buckhout et al., 2009; Garcia et al., 2010; Yang et al., 2010). Biological roles of these differentially expressed PKs and PPs, however, cannot be inferred solely based on the transcript level without functional characterization by genetic approaches. None the less, studying hundreds of differentially expressed genes without any selection filter would be extremely laborious. Co-expression analysis provides a way to filter and select genes of interest for the biological question under study (Ihmels et al., 2004; Kharchenko et al., 2005). The expression of genes within the same metabolic pathway shows often similar pattern; thus, co-expression analysis can aid in discovering upstream regulators or downstream substrates of a particular metabolic pathways (Ihmels et al., 2004; Kharchenko et al., 2005).

In order to gain insights into the regulation of the responses to Fe deficiency at the post-translational level, we analyzed the expression of PK and PP genes in Fe-deficient roots using the RNA-seq technology. Genes encoding PKs and PPs that were differentially expressed upon Fe starvation were clustered into groups of closely correlated modules based on their co-expression relationships under various sets of experimental conditions. Using this approach, we discovered PKs with potentially critical regulatory functions in cellular Fe and potassium (K) ion homeostasis under Fe-deficient conditions.

## Results

### Expression of PK and PP genes in Fe-deficient *Arabidopsis* roots

**Figure 1:**
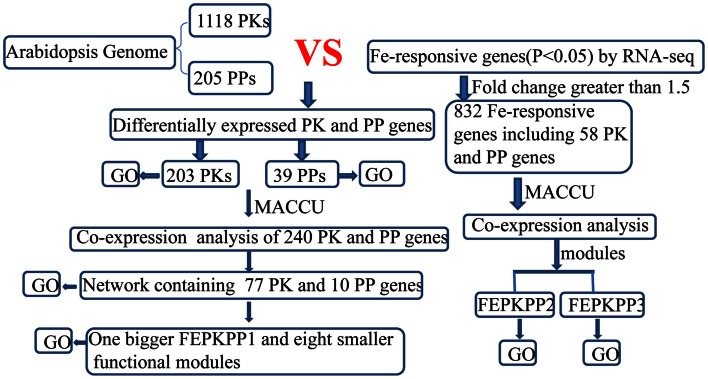
**Flowchart for mining differentially expressed PK and PP genes and subsequent co-expression analysis in *Arabidopsis* iron-deficient roots**.

**Figure 2:**
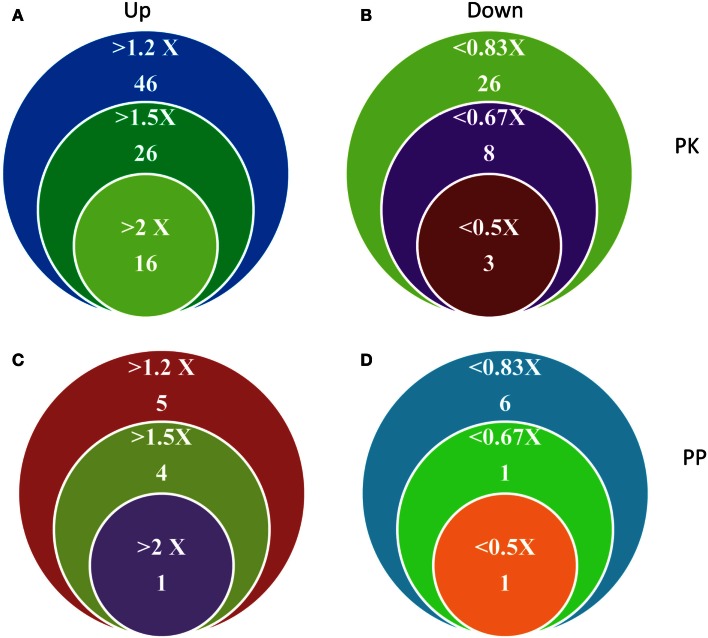
**Differentially expressed PK and PP genes in Fe-deficient *Arabidopsis* roots**. **(A,B)** Number and expression levels of PK genes. **(C,D)** Number and expression levels of PP genes.

Transcriptional changes in the expression of PK and PP genes upon Fe deficiency in *Arabidopsis* roots were mined in a previously published RNA-seq data set (Li et al., submitted). The flowchart was shown as Figure 1. Out of 1,118 PK (GO: 0004672) and 205 PP genes (GO: 0004721) annotated in the TAIR10 release of the *Arabidopsis* genome, 203 PK and 39 PP genes were differentially expressed between Fe-sufficient and Fe-deficient plants (*P* < 0.05; Table S1 in Supplementary Material). Among the 203 PK genes, 88 and 37 genes were induced and repressed by Fe deficiency with fold-changes greater than 1.2 (Figures 2A,B), transcripts of 53 genes were changed more than 1.5-fold upon Fe deficiency (Table 1). Interestingly, 38 out of these 53 genes belong to the receptor-like kinase (RLK) and receptor-like cytoplasmic protein kinase (RLCK) superfamily (Table 1). The second most predominant group (eight genes) encodes PKs from the CAMK_AMPK/CDPK subfamily (Table 1). Among the 39 differentially expressed PP genes, 18 genes were up- or down-regulated by Fe deficiency with fold-changes greater than 1.2 (Figures 2C,D), transcripts of seven genes were changed more than 1.5-fold upon Fe deficiency (Table 2). Two genes, At2g46700 and At3g49370, are annotated to possess both PK and PP activity and are listed in both Tables 1 and 2.

**Table 1:** **Differentially expressed protein kinase genes upon iron deficiency with fold change of more than 1.5-fold**.

AGI	Function	Mean (−Fe/+Fe)	SD	Subfamily
AT2G19410	Protein kinase family protein	10.26	0.95	RLCK
AT5G53450	ORG1 (OBP3-responsive gene 1); ATP binding/kinase/protein kinase	5.24	0.18	
AT5G01060	Protein kinase family protein	5.01	0.85	RLCK
AT1G51870	Protein kinase family protein	4.10	2.00	RLK
AT1G77280	Protein kinase family protein	4.09	0.46	RLCK
AT4G38830	Protein kinase family protein	3.54	0.49	RLK
AT1G16120	WAKL1 (wall associated kinase-like 1)	2.76	0.57	RLK
AT5G39000	protein kinase family protein	2.75	0.63	RLK
AT1G16150	WAKL4 (WALL ASSOCIATED KINASE-LIKE 4)	2.72	0.39	RLK
AT5G23170	Protein kinase family protein	2.62	0.40	RLCK
AT1G05700	Leucine-rich repeat protein kinase	2.60	0.40	RLK
AT1G51830	ATP binding/kinase/protein serine/threonine kinase	2.58	0.19	RLK
AT4G26890	MAPKKK16; ATP binding/kinase/protein kinase/protein serine/threonine kinase/protein tyrosine kinase	2.18	0.13	Group-C
AT5G07280	EMS1 (EXCESS MICROSPOROCYTES1); kinase/transmembrane receptor protein kinase	2.14	0.27	RLK
AT1G33260	Protein kinase family protein	2.03	0.19	RLCK
AT1G51860	Leucine-rich repeat protein kinase	2.02	0.32	RLK
AT1G72540	Protein kinase, putative	1.97	0.33	RLCK
AT2G28990	Leucine-rich repeat protein kinase	1.96	0.08	RLK
AT5G60280	Lectin protein kinase family protein	1.93	0.36	RLK
AT4G18700	CIPK12 (CBL-INTERACTING PROTEIN KINASE 12); ATP binding/kinase/protein kinase/protein serine/threonine kinase	1.91	0.12	CAMK_AMPK
AT2G45590	Protein kinase family protein	1.89	0.32	RLCK
AT2G30360	SIP4 (SOS3-INTERACTING PROTEIN 4); kinase/protein kinase	1.89	0.21	CAMK_CDPK
AT3G46330	MEE39 (maternal effect embryo arrest 39)	1.78	0.28	RLK
AT1G51620	Protein kinase family protein	1.78	0.55	RLCK
AT1G08650	PPCK1 (PHOSPHOENOLPYRUVATE CARBOXYLASE KINASE); kinase/protein serine/threonine kinase	1.78	0.16	CAMK_CDPK
AT5G35580	ATP binding/kinase/protein kinase/protein serine/threonine kinase/protein tyrosine kinase	1.73	0.44	RLCK
AT3G49370	CDPK-related kinase	1.71	0.30	CAMK_AMPK
AT1G01140	CIPK9 (CBL-INTERACTING PROTEIN KINASE 9); ATP binding/kinase/protein kinase/protein serine/threonine kinase	1.71	0.37	CAMK_CDPK
AT3G27580	ATPK7; kinase/protein serine/threonine kinase	1.69	0.21	AGC_S6K
AT1G16160	WAKL5 (wall associated kinase-like 5)	1.64	0.34	RLK
AT3G45330	Lectin protein kinase family protein	1.62	0.19	RLK
AT5G55560	Protein kinase family protein	1.62	0.34	Other_WNK
AT1G07560	Leucine-rich repeat protein kinase	1.61	0.21	RLK
AT3G57740	Protein kinase family protein	1.61	0.39	RLCK
AT5G25440	Protein kinase family protein	1.59	0.16	RLCK
AT1G51800	Leucine-rich repeat protein kinase	1.58	0.12	RLK
AT1G66930	Serine/threonine protein kinase family protein	1.57	0.15	RLK
AT1G74360	Leucine-rich repeat transmembrane protein kinase	1.56	0.22	RLK
AT5G16900	Leucine-rich repeat protein kinase	1.56	0.15	RLK
AT4G04700	CPK27; ATP binding/calcium ion binding/kinase/protein kinase/protein serine/threonine kinase/protein tyrosine kinase	1.56	0.16	CAMK_CDPK
AT5G35750	AHK2 (*ARABIDOPSIS* HISTIDINE KINASE 2); cytokinin receptor/osmosensor/protein histidine kinase	1.56	0.03	
AT2G46700	CDPK-related kinase	1.52	0.11	CAMK_AMPK
AT3G50230	Leucine-rich repeat transmembrane protein kinase	0.64	0.09	RLK
AT5G49760	Leucine-rich repeat family protein/protein kinase family protein	0.63	0.04	RLK
AT1G61480	S-locus protein kinase, putative	0.62	0.04	RLK
AT5G49780	ATP binding/kinase/protein serine/threonine kinase	0.62	0.03	RLK
AT2G25090	CIPK16 (CBL-INTERACTING PROTEIN KINASE 16); ATP binding/kinase/protein kinase/protein serine/threonine kinase	0.61	0.09	CAMK_AMPK
AT5G59660	Leucine-rich repeat protein kinase	0.60	0.05	RLK
AT1G07150	MAPKKK13; ATP binding/kinase/protein kinase/protein serine/threonine kinase	0.60	0.13	Group-C
AT2G18470	Protein kinase family protein	0.53	0.11	RLK
AT1G74490	Protein kinase, putative	0.46	0.09	RLCK
AT1G21230	WAK5 (WALL ASSOCIATED KINASE 5)	0.44	0.14	RLK
AT4G40010	SNRK2.7 (SNF1-RELATED PROTEIN KINASE 2.7)	0.44	0.08	Group-A

*The corresponding mean ratios, defined as the transcript level (Reads Per Kilobase per Million mapped reads) in the –Fe treatment divided by the level in the +Fe treatment (*P* < 0.05)*.

**Table 2:** **Differentially expressed protein phosphatase genes upon iron deficiency with fold change of more than 1.5-fold**.

AGI	Function	Mean (−Fe/+Fe)	SD	Alias
AT2G01880	PAP7 (PURPLE ACID PHOSPHATASE 7); acid phosphatase/protein serine/threonine phosphatase	3.11	0.42	PAP7
AT2G32960	Tyrosine specific protein phosphatase family protein	1.95	0.19	PFA-DSP2
AT3G49370	CDPK-related kinase	1.71	0.30	
AT2G01890	PAP8 (PURPLE ACID PHOSPHATASE 8); acid phosphatase/protein serine/threonine phosphatase	1.57	0.04	PAP8
AT2G46700	CDPK-related kinase	1.52	0.11	CRK3
AT5G26010	Catalytic/protein serine/threonine phosphatase	0.65	0.07	
AT5G59220	Protein phosphatase 2C, putative/PP2C, putative	0.45	0.04	SAG113

*The corresponding mean ratios, defined as the transcript level (Reads Per Kilobase per Million mapped reads) in the −Fe treatment divided by the level in the +Fe treatment (*P* < 0.05)*.

### Gene ontology enrichment analysis of the differentially expressed PK and PP genes

Gene Ontology (GO) enrichment analysis revealed that the products of most of the 203 PKs localizes to the plasma membrane, the endomembrane system, and the micropyle (inset of Figure S1 in Supplementary Material, *P* < 0.01), and are involved in diverse biological processes (*P* < 0.01, Figure S1 in Supplementary Material). To gain insights into the physiological roles of the differentially expressed PKs, a GO enrichment analysis of PK genes that showed expression changes of more than 1.5-fold was performed. The results showed that that the categories “regulation of potassium ion transport,” “tapetal cell fate specification,” “response to nickel ion,” and “cellular response to potassium ion starvation” were overrepresented (Figure S2 in Supplementary Material). Most of the products of these genes are localized in the endomembrane system.

Among the 39 differentially expressed PP genes, the processes “hypotonic salinity response,” “photosystem stoichiometry adjustment,” and “cellular potassium ion homeostasis” etc. were enriched (Figure S3A in Supplementary Material). Gene products are localized in the protein serine/threonine phosphatase complex, on the plasma membrane, and in the calcineurin complex (Figure S3B in Supplementary Material). Within the seven PP genes with more than 1.5-fold change, none of the cellular components or biological processes was enriched.

### CO-expression analysis of Fe-responsive PK and PP genes

**Figure 3:**
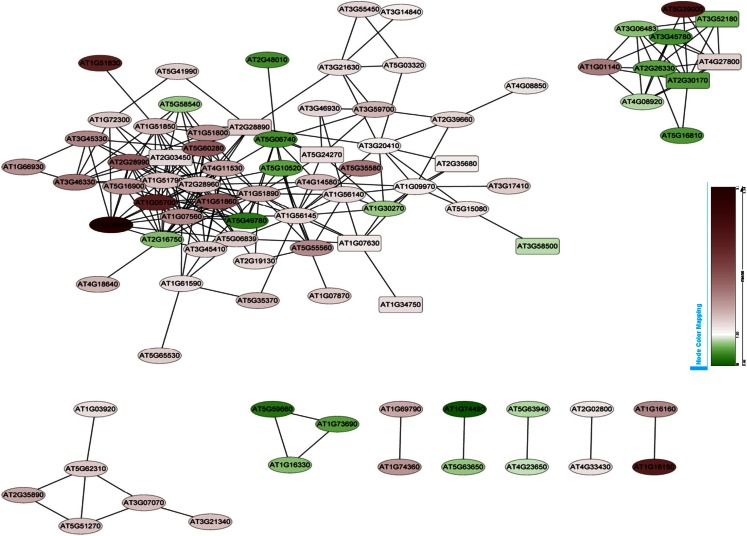
**Co-expression relationships of the differentially expressed PK and PP genes**. Red nodes indicate up-regulated genes, green nodes denote genes that are repressed by Fe deficiency. Round-shaped nodes represent protein kinase genes, rectangles indicate protein phosphatase genes.

**Figure 4:**
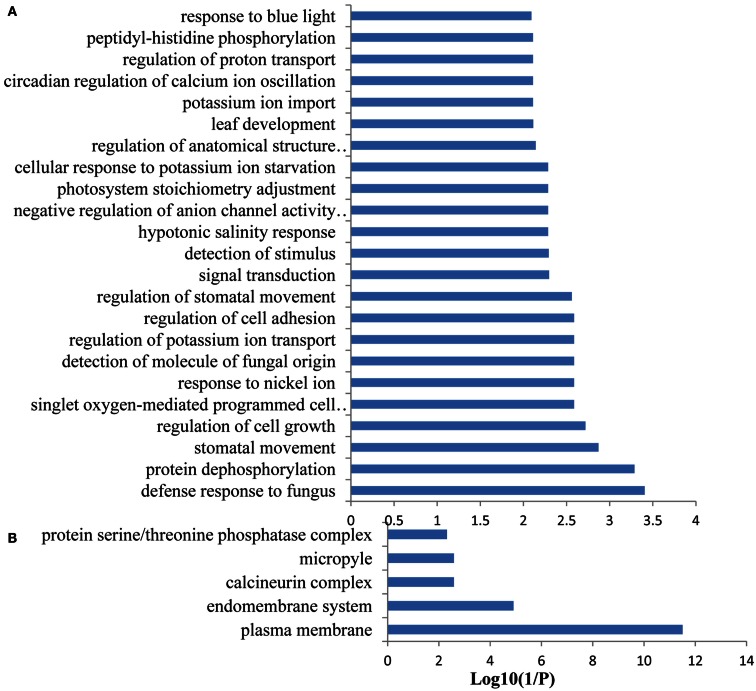
**Gene ontology (GO) enrichment analysis of the genes forming the network**. GO enrichment of the 87 PK and PP genes which involved in the co-expression network was performed with the Gene Ontology Browsing Utility (GOBU) (Lin et al., 2006) using the TopGo “elim” method (Alexa et al., 2006) with *P* < 0.01. **(A)** GO of the biological process and **(B)** GO of the subcellular localization.

**Figure 5:**
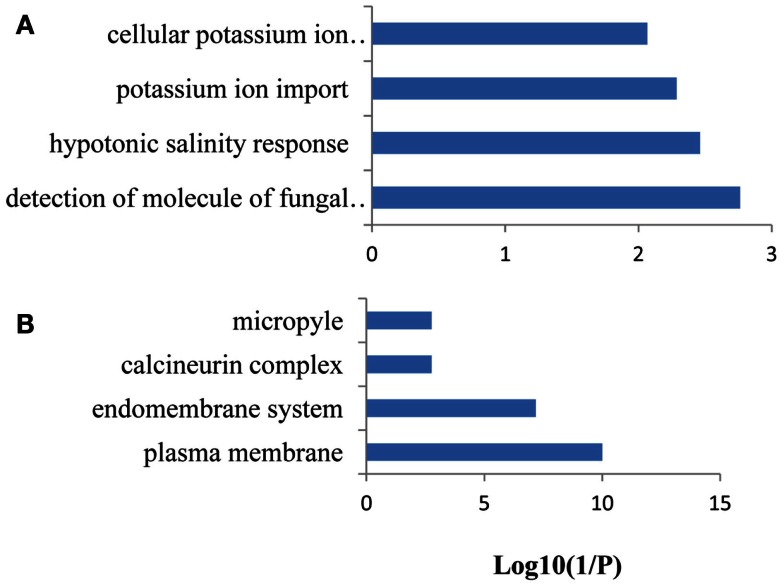
**Gene ontology (GO) enrichment analysis of the genes from FEPKPP1**. GO enrichment of the 58 PK and PP genes in the module FEPKPP1 was performed with the GOBU toolbox using the TopGo “elim” method with *P* < 0.01. **(A)** GO biological process and **(B)** GO subcellular localization.

Co-expression networks of differentially expressed PK and PP genes were generated using the MACCU software (Lin et al., 2011). Pairwise co-expressed genes were selected with a Pearson correlation coefficient cutoff of 0.7 (Lin et al., 2011; Wang et al., 2012). The 300 publicly available microarrays that were mined for analyzing co-expression relationship discriminated root-related experiments and the co-expression relationships reported here are restricted to roots. Because protein phosphorylation is reversible, both PK and PP genes were used to generate the network. The total number of differentially expressed PK and PP genes was 240. Co-expression relationships between these genes were visualized using Cytoscape^1^. The network of PK and PP genes responsive to Fe deficiency consists of 87 nodes (77 PKs genes and 10 PPs genes) and 248 edges (correlations between genes; Figure 3). The network can be further divided into one larger and eight smaller clusters. GO enrichment analysis revealed that the biological processes “defense response to fungus,” “stomatal movement,” “regulation of cell growth,” and “response to nickel ion” were most strongly enriched (Figure 4A), the products of these genes were chiefly localized on the plasma membrane, in the endomembrane system, in the calcineurin complex, in the micropyle, and in the protein serine/threonine phosphatase complex (Figure 4B). The largest module of the network, named FEPKPP1, consists of 51 PK and seven PP genes (Table S2 in Supplementary Material). More than 76% of the genes in this module belong to the RLK superfamily, including 33 RLKs and 11 RLCKs. GO analysis showed that genes involved in the biological processes “detection of molecule of fungal origin,” “hypotonic salinity response,” and “potassium ion import and cellular potassium ion homeostasis,” and the localizations “plasma membrane,” “micropyle,” and “calcineurin complex” were enriched in this cluster (Figures 5A,B).

Co-expression analysis of the 58 differentially expressed PK and PP genes with fold-changes greater than 1.5 using the criteria mentioned above yielded a network consisting of 14 nodes and 32 edges; none of the PP genes was associated with the network (Figure S4 in Supplementary Material). The network can be divided into one larger and one smaller cluster. GO enrichment analysis of the bigger module revealed that, similar to cluster FEPKPP1, the biological processes “detection of molecule of fungal origin,” “hypotonic salinity response,” and “potassium ion import and cellular potassium ion homeostasis” were enriched.

### A genome-wide functional network associated with Fe-responsive PK and PP genes

**Figure 6:**
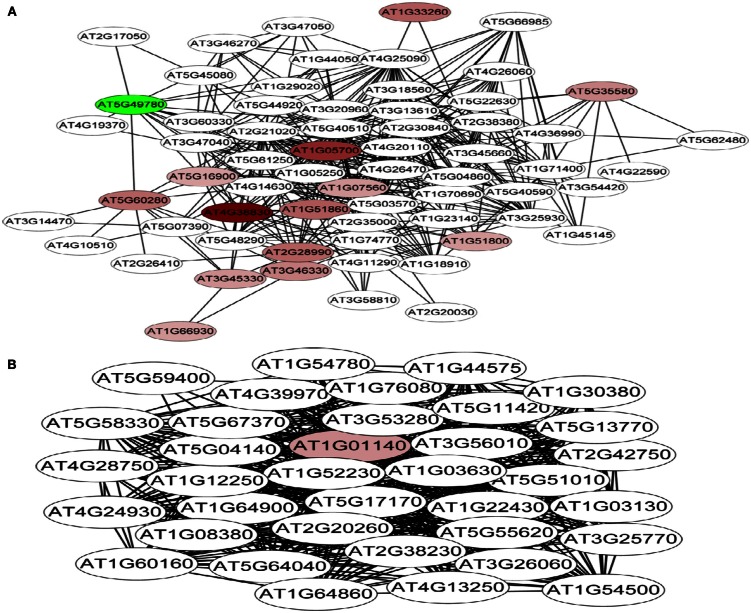
**Co-expression relationships of Fe-responsive genes with fold-changes greater than 1.5-fold in *Arabidopsis* roots**. **(A)** Module FEPKPP2 and **(B)** module FEPKPP3. Red nodes indicate up-regulated PK genes, green nodes denote PK genes that are repressed by Fe deficiency, white nodes indicate fished genes.

**Figure 7:**
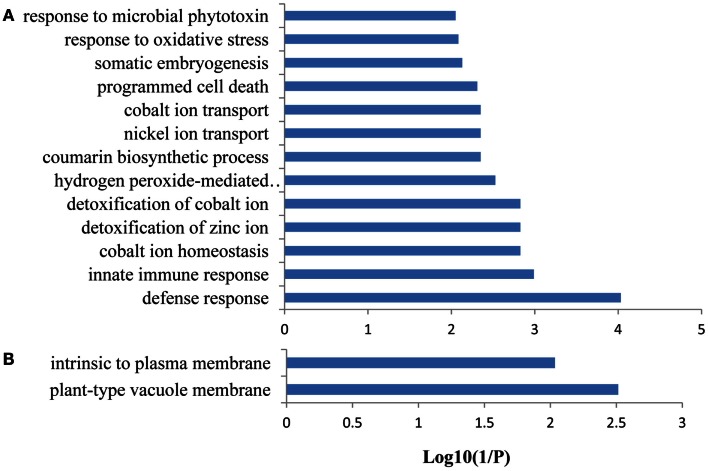
**Gene ontology (GO) enrichment analysis of the genes from FEPKPP2**. GO enrichment of the 64 Fe-responsive genes in the module FEPKPP2 was performed with the GOBU toolbox using the TopGo “elim” method with *P* < 0.01. **(A)** GO biological process and **(B)** GO subcellular localization.

**Figure 8:**
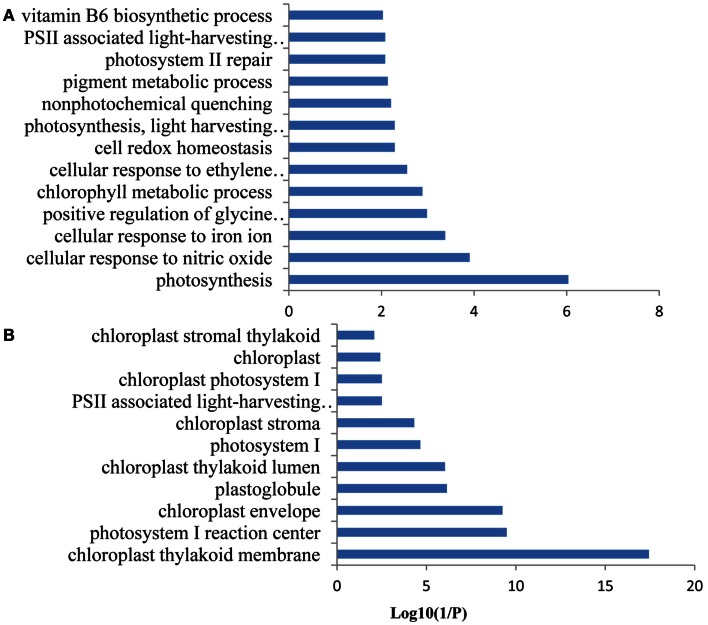
**Gene ontology (GO) enrichment analysis of the genes from FEPKPP3**. GO enrichment of the 36 Fe-responsive genes in the module FEPKPP3 was performed with the GOBU toolbox using the TopGo “elim” method with *P* < 0.01. **(A)** GO biological process and **(B)** GO subcellular localization.

A co-expression network of 832 Fe-responsive genes including 58 PK and PP genes with fold-changes greater than 1.5 was generated as described above. In the network shown in Figure 6 only nodes that were not connected by at least one edge to a bait (PK or PP) gene and edges linked to two preys (all other differentially expressed genes) were excluded. Also this network could be divided into two clusters, one of which comprises 64 nodes and 440 edges with 14 PK genes (Figure 6A, herein named FEPKPP2), and a smaller cluster consisting of 36 nodes and 564 edges with only one PK genes (Figure 6B, herein named FEPKPP3). The majority of the prey genes in this cluster were induced by Fe starvation; only transcripts derived from five out of 50 prey genes showed decreased abundance (Table S3 in Supplementary Material). None of the products of these prey genes are identified as Fe-responsive phosphoproteins (Lan et al., 2012b), possibly due to most of these products are membrane proteins not easy to be detected by phosphoproteomics. GO enrichment analysis of the module FEPKPP2 revealed that the biological processes “defense response,” “innate immune response,” and “cobalt ion transport and cellular homeostasis” were enriched (Figure 7A), with the majority of the gene products localized to the tonoplast or intrinsic to the plasma membrane (Figure 7B). This stands in contrast to the genes in the module FEPKPP3 where 30 out of the 35 prey genes showed decreased transcript abundance upon Fe deficiency (Table S4 in Supplementary Material). GO enrichment analysis of this module revealed that the biological processes “photosynthesis,” “cellular response to nitric oxide,” “cellular response to iron ion,” “cell redox homeostasis,” “photosystem II repair,” and “vitamin B6 biosynthetic process” were enriched (Figure 8A). The gene products mainly localized to the chloroplast thylakoid membrane, photosystem I reaction center, the chloroplast envelope, plastoglobules, the chloroplast thylakoid lumen, and the chloroplast stroma (Figure 8B).

## Discussion

The possible function of post-translational modifications of proteins in the Fe-deficiency response remains poorly understood. PKs and PPs play key roles in the regulation of nearly all aspects of metabolism and development. Due to the use of microarray probe sets that have significant cross hybridization potential and are unable to distinguish highly similar genes of this subfamily, transcriptional information on the expression of PK and PP genes in response to Fe deficiency is lacking in *Arabidopsis*. The RNA-seq technology has proven to provide precise “digital” information on gene expression, and is able to discriminate genes of high sequence identity (Ozsolak and Milos, 2011). Using this technology, we here present comprehensive information on the transcriptional expression of PK and PP genes in Fe-deficient *Arabidopsis* roots. Based on our criteria, less than 20% of the annotated PK and PP genes were differentially expressed upon Fe deficiency and only around 5% PK and PP genes were differentially expressed with fold-changes greater than 1.5. GO enrichment analysis of the 203 differentially expressed PK genes revealed that these PKs were involved in various biological processes (Figure S1 in Supplementary Material). Considering only the 53 PK genes with fold-changes greater than 1.5, the GO categories “regulation of potassium ion transport,” “tapetal cell fate specification,” “response to nickel ion,” and “cellular response to potassium ion starvation” were overrepresented (Figure S2 in Supplementary Material). The subtle transcriptional response of these genes suggests that under conditions of Fe-deficiency protein phosphorylation may function to fine-tune basic housekeeping processes. Alternatively, transcription is not usually the main level for regulation of PK and PP activities, which occurs mostly at the post-translational level. The shift in overrepresented GO categories in robustly up-regulated PK genes may indicate post-translational regulation of processes that are critical for cellular Fe homeostasis such as potassium ion transport and response to potassium and nickel ions. Interestingly, 87% of the robustly regulated PK genes belong to the RLK/RLCK and CAMK_AMPK/CDPK subfamilies (Table 1), implicating that PKs from these families may be important for adaptation to Fe deficiency in *Arabidopsis* roots. This finding is consistent with our previous quantitative phosphoproteomic study that predicted two pro-directed kinases [A protein kinase that phosphorylates certain Ser/Thr residues that precede a Pro residue (Ser/Thr-Pro motifs) (Lu and Zhou, 2007)] belonging to RLK superfamily to play critical roles in regulating Fe-responsive phosphopeptides (Lan et al., 2012b). RLKs are defined by the presence of a signal peptide, an extracellular domain, which is absent in the RLCK subfamily, a transmembrane domain region that anchors the receptor in the cell membrane, and a carboxyl-terminal serine/threonine kinase domain (Wang et al., 2007). More than 2% of the predicted *Arabidopsis* coding sequences encode RLKs, which have diverse functions in development, pathogen resistance, hormone perception, and environmental adaption (Wang et al., 2007).

The strong overrepresentation of PK and PP genes encoding proteins involved in potassium homeostasis was an unexpected result. The link between potassium uptake and Fe deficiency remains elusive. One possible explanation is that PKs are required in the regulation of both potassium and Fe homeostasis. Some PKs may play broader roles in nutrient signaling. For example, CIPK23 was reported to be required for both nitrate sensing and activation of the potassium channel AKT1 (Xu et al., 2006; Ho et al., 2009). Alternatively, there might be undiscovered cross-talks between Fe and K deficiency signaling. Evidence for such cross-talk has been inferred from microarray analysis that revealed that the Fe transporter *LeIRT1* is up-regulated by K deficiency (Wang et al., 2002). Moreover, expression of the K transporter gene *LeKC1* was strongly increased by Fe deficiency and K starvation, further supporting such a scenario (Wang et al., 2002). An ameliorating effect of K supply on Fe deficiency has been described three decades ago (Barak and Chen, 1983), and was associated with a change in the cation/anion uptake balance.

Genes showing similar expression pattern under diverse conditions often have correlative functions (Eisen et al., 1998), and the processes in which genes with unknown functions are involved can be inferred from their co-expression relationships with genes with known functions (Aoki et al., 2007; Usadel et al., 2009). In the present study, the global expression of PK and PP genes in *Arabidopsis* roots was analyzed to gain insights into the interplay of transcriptional responses to Fe deficiency. By mining public databases, PK and PP genes that were differentially expressed upon Fe starvation were clustered into groups of closely correlated modules based on their co-expression relationships under various sets of experimental conditions. Using this approach, we discovered nine potentially critical regulatory modules with various putative roles under Fe deficiency (Figures 3 and 4). Interestingly, only 35% of the differentially expressed PK and PP genes were constituents of the co-expression network, suggesting that the majority of PKs and PPs responsive to Fe deficiency are functionally diverse and involved in a variety of biological processes and metabolic pathways. It is noteworthy that, compared to the 26% of the differentially expressed PP genes that are involved in the network, the group of PKs is clearly better represented (38% of the differentially expressed PK genes are included in the network), even though the percentage of differentially expressed PP gene is slightly higher than that of PK genes (39 out of 205 PP genes VS 203 out of 1,118 PK genes). Some modules contain only a few or no PP genes at all (Figure 2). These observations support the assumption that the regulation of biological processes requires a cascaded and/or coordinated protein phosphorylation by different PKs to adapt to environmental stresses, while the PP-mediated removal of phosphate from a phosphorylated protein is less specific.

To explore potential upstream regulators and downstream targets of the PK and PP genes, a co-expression network was constructed from the 774 Fe-responsive genes (excluding PK and PP genes) with fold change greater than 1.5-fold and the 58 PK and PP genes as baits. Co-expression network generated in this study was mainly associated with PK and PP genes, which is different from those previously reported gene co-expression networks where they consider the global Fe supply dependent co-expression networks in *Arabidopsis* roots (Schmidt and Buckhout, 2011; Ivanov et al., 2012). This network could be divided into two sub-modules, named FEPKPP2 and FEPKPP3 (Figure 6). Interestingly, in the module FEPKPP2 90% of the prey genes were induced by Fe deficiency while the transcript abundance of 86% prey genes in module FEPKPP3 were decreased (Tables S3 and S4 in Supplementary Material). Products of most prey genes in FEPKPP2 are localized on the tonoplast and on the plasma membrane. Several transporters, such as the Co, Ni, and Fe ion transporter IREG2 (Schaaf et al., 2006; Morrissey et al., 2009), the Zn detoxification protein MPT3 (Arrivault et al., 2006), and the proton-translocating ATPase AHA7 were induced upon Fe deficiency and were tightly co-expressed with PKs. For example, IREG2 was directly co-expressed with the PKs At4g38830 (ATMPK8), At5g16900 (ATMPK9), At3g46330, and At1g07560, indicating that these PKs might be required for IREG2 regulation. MPT3 was directly co-expressed with the LRR subfamily PKs At1g07560 and At1g51860, suggesting that these PKs play major roles in detoxification of excess zinc by MPT3. AHA7, reported to be involved in Fe-deficiency-induced root hair formation (Santi and Schmidt, 2009), was directly co-expressed with ATMPK8, indicating that ATMPK8-mediated AHA7 phosphorylation might be critical for the morphological changes in Fe-deficient roots. The module FEPKPP3 is composed of 36 genes, with only one PK gene, *CIPK9*, closely co-expressed with the prey genes. This suggests that CIPK9 has broad substrate specificity and is involved in diverse processes. A recent study has shown that CIPK9 is involved in K homeostasis under low K stress (Liu et al., 2013). *CIPK9* was induced by Fe deficiency but the majority of the prey genes were repressed by Fe starvation. For example, seven photosynthesis-related prey genes (At1g54780, At2g20260, At1g52230, At1g03130, At5g64040, At1g08380, and At1g30380) that were co-expressed with *CIPK9* showed decreased transcript abundance upon by Fe deficiency. Photosynthesis has been shown to be down-regulated in response to Fe deficiency also in leaves and, unexpectedly, also in roots (Buckhout et al., 2009), indicating that this is a conserved component of the Fe-deficiency response that may be implicated in Fe signaling pathways. Alternatively, some of the photosynthesis-related genes may have acquired novel functions in roots that are associated with the re-calibration of cellular Fe homeostasis. GO enrichment analysis of this module also revealed that the biological processes “cellular response to nitric oxide” and “cellular response to iron ion” were enriched (Figure 8A), due to two genes At5g55620 and At3g53280 in this module. These two genes are strongly induced by both nitric oxide treatment and Fe deficiency (Garcia et al., 2010).

## Conclusion

In summary, we provide genome-wide information on the transcriptional expression of PK and PP genes in Fe-deficient *Arabidopsis* roots and on the biological processes putatively controlled by reversible phosphorylation. A root-specific co-expression network of Fe-responsive genes encoding PKs and PPs predicted ATMPK8, ATMPK9, and CIPK9 as putative novel players in the control of cellular Fe homeostasis. The results further show that the control of ion transport across the plasma membrane and the vacuolar membrane as well as plastid development and function PK are dependent. The approach applied here will be useful to direct further studies and to decipher the mechanisms by which ion transporters and plastid function is controlled post-translationally in Fe-deficient plants.

## Materials and Methods

### Methods

#### Data collection and processing

Transcriptome data of roots from 13-day-old *Arabidopsis* seedlings grown in the presence or absence of Fe by RNA-seq were downloaded from a public database (NCBI: SRA045009) and analyzed as described in (Lan et al., 2012a). Microarray data of 2,671 ATH1 arrays from the NASCarray database^2^ were downloaded and normalized using the RMA function of the Affy package of the Bioconductor software. Three hundred root-related arrays were manually identified as described in (Lin et al., 2011), and were used as a data base for co-expression analysis. PK and PP genes were retrieved on the basis of the TAIR 10 release of *Arabidopsis* genome.

#### Gene ontology analysis

Enrichment analysis of GO categories was performed with the Gene Ontology Browsing Utility (GOBU) (Lin et al., 2006) using the TopGo “elim” method (Alexa et al., 2006) from the aspects of “biological process” and “subcellular localization.” The elim algorithm iteratively removes the genes mapped to significant terms from higher level GO terms, and thus avoids unimportant functional categories being enriched.

### Generation of co-expression networks and modules of Fe-responsive PK and PP genes using the MACCU toolbox

To generate root-specific networks of Fe-responsive PK and PP genes, differentially expressed PK and PP genes were identified using a Student *t*-test at a *P* < 0.05. Gene networks were constructed based on 300 publicly available root-related microarrays using the MACCU toolbox as described in (Lin et al., 2011), with a Pearson correlation threshold of 0.7. The generated co-expression networks were visualized by Cytoscape (see text footnote 1). If one cluster of genes did not have any connection (edges) to any other cluster in the co-expression network, we referred to such a cluster as a module.

### Construction of Fe-responsive root networks with PK and PP genes

To obtain root-related, Fe-responsive gene networks comprising PK and PP genes, subsets of 774 Fe-responsive genes with fold-changes greater than 1.5-fold were mined. Next, 58 Fe-responsive PK and PP genes were extracted (bait genes), combined with the root Fe-responsive genes (preys), and used for generating co-expression network. The resulting networks show only those nodes (genes) and edges (relationships between genes) that were linked by at least one edge with a bait gene. Edges linked to two preys were excluded.

## Conflict of Interest Statement

The authors declare that the research was conducted in the absence of any commercial or financial relationships that could be construed as a potential conflict of interest.

## Supplementary Material

The Supplementary Material for this article can be found online at: http://www.frontiersin.org/Plant_Nutrition/10.3389/fpls.2013.00173/abstract

Table S1 **Differentially expressed protein kinase and phosphatase genes upon iron deficiency**. The corresponding response ratios, defined as the transcript level (Reads Per Kilobase per Million mapped reads) in the −Fe treatment divided by the level in the +Fe treatment, are shown in three biological repeats, as well as the mean (*P* < 0.05).

Table S2 **Genes associated with the major module FEPKPP1**.

Table S3 **Genes associated with the module FEPKPP2**.

Table S4 **Genes associated with the module FEPKPP3**.

Figure S1 **Gene ontology (GO) enrichment analysis of the 203 differentially expressed PK genes**. GO enrichment of the “biological process” of the 203 Fe-responsive PK genes was performed with the GOBU toolbox using the TopGo “elim” method with *P* < 0.01. Inset indicates GO of the subcellular localization.

Figure S2 **Gene ontology of 53 PK genes with fold-changes greater than 1.5-fold**. GO enrichment of the 53 Fe-responsive PK genes with fold-changes greater than 1.5-fold was performed with the GOBU toolbox using the TopGo “elim” method with *P* < 0.01.

Figure S3 **Gene ontology of the 39 differentially expressed PP genes**. GO enrichment of the 39 Fe-responsive PP genes was performed with the GOBU toolbox using the TopGo “elim” method with *P* < 0.01. **(A)** GO biological process and **(B)** GO subcellular localization.

Figure S3 **Co-expression relationships of Fe-responsive PK and PP genes with fold-changes greater 1.5-fold in *Arabidopsis* roots**.

## Footnotes

^1^http://www.cytoscape.org

^2^http://affymetrix.arabidopsis.info/
