# Supplementary material for: A Digital Compendium of Genes Mediating the Reversible Phosphorylation of Proteins in Fe-Deficient Arabidopsis Roots
Source: Front Plant Sci. 2013 Jun 3;4:173. doi: 10.3389/fpls.2013.00173 (PMC3669753; doi:10.3389/fpls.2013.00173)
Supplement: Table S1 — Differentially expressed protein kinase and phosphatase genes upon iron deficiency. The corresponding response ratios, defined as the transcript level (Reads Per Kilobase per Million mapped reads) in the −Fe treatment divided by the level in the +Fe treatment, are shown in three biological repeats, as well as the mean (P < 0.05). [file 52747_Lan_DataSheet1.ZIP › 52747_Lan_Supplementary_Figures.pdf]

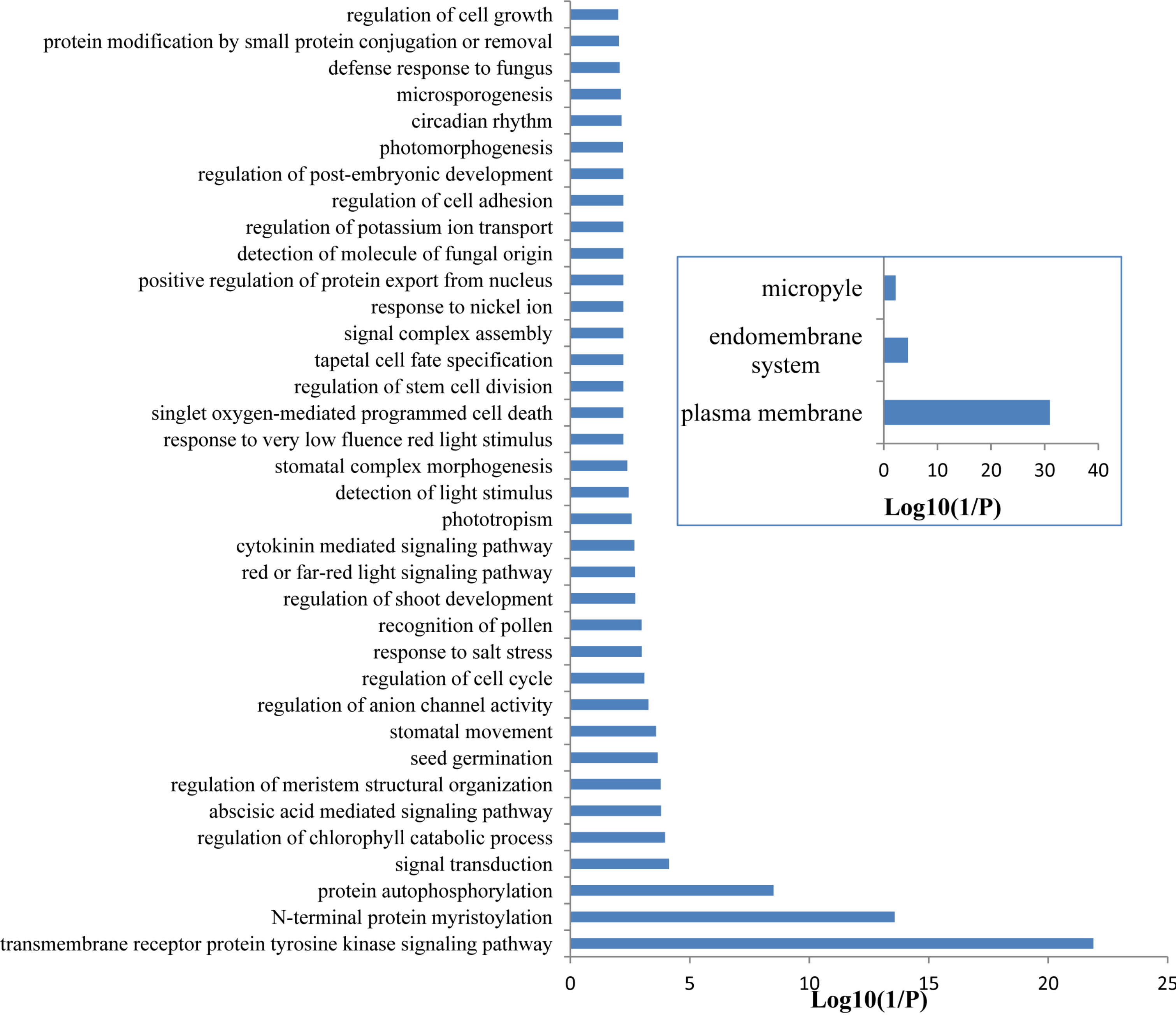

Figure A1-GO of 203 Fe-responsive PK

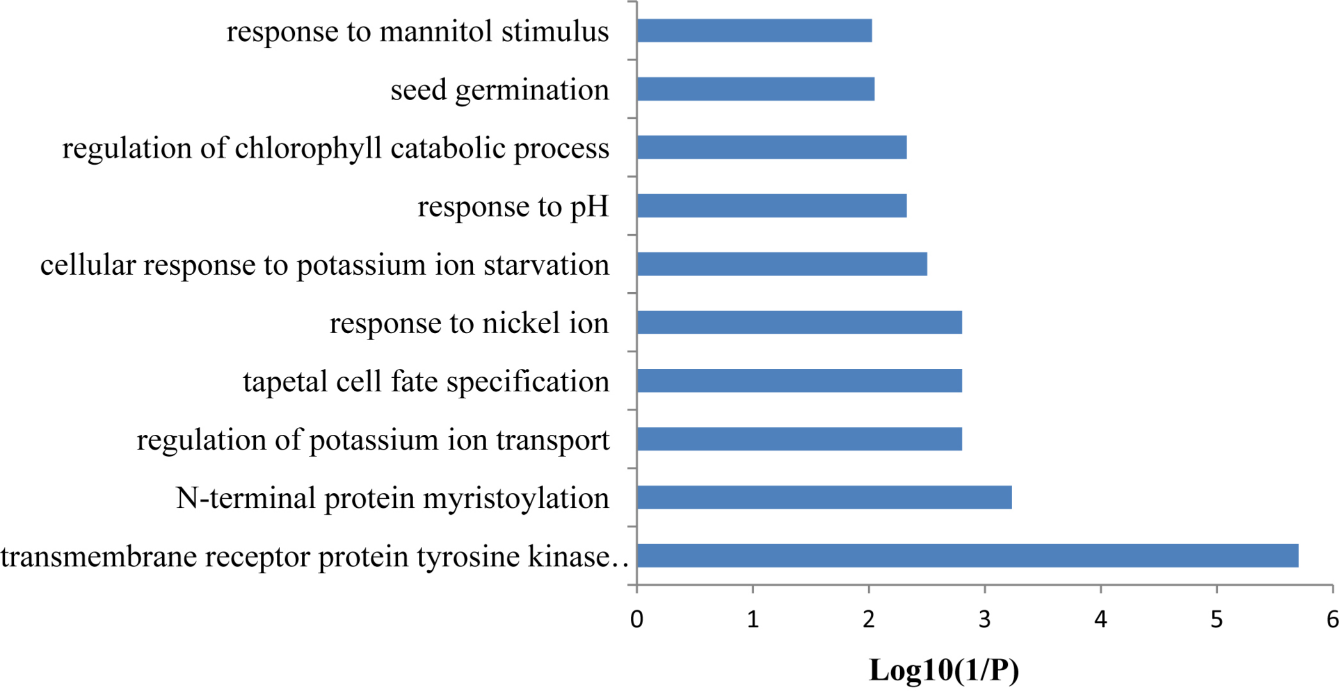

Figure A2-GO of 53 PK genes with fold change of more than 1.5 fold

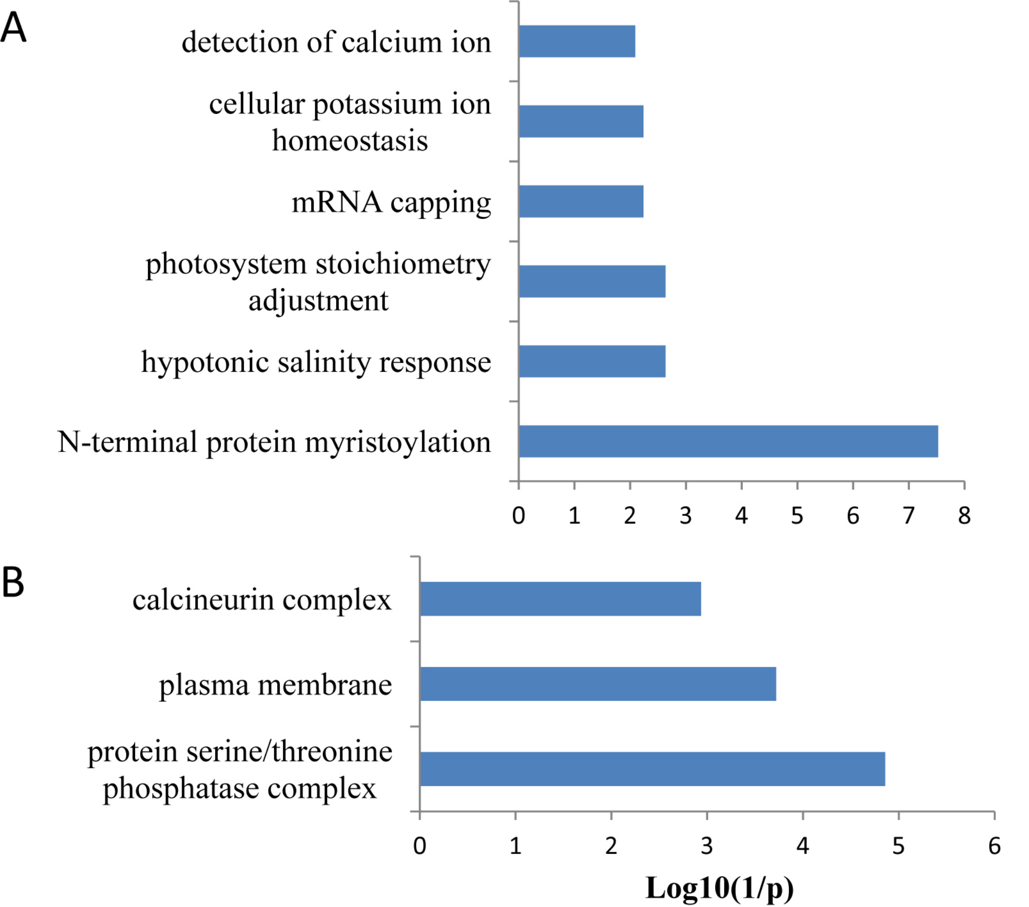

Figure A3-GO of the 39 differentially expressed PP genes.

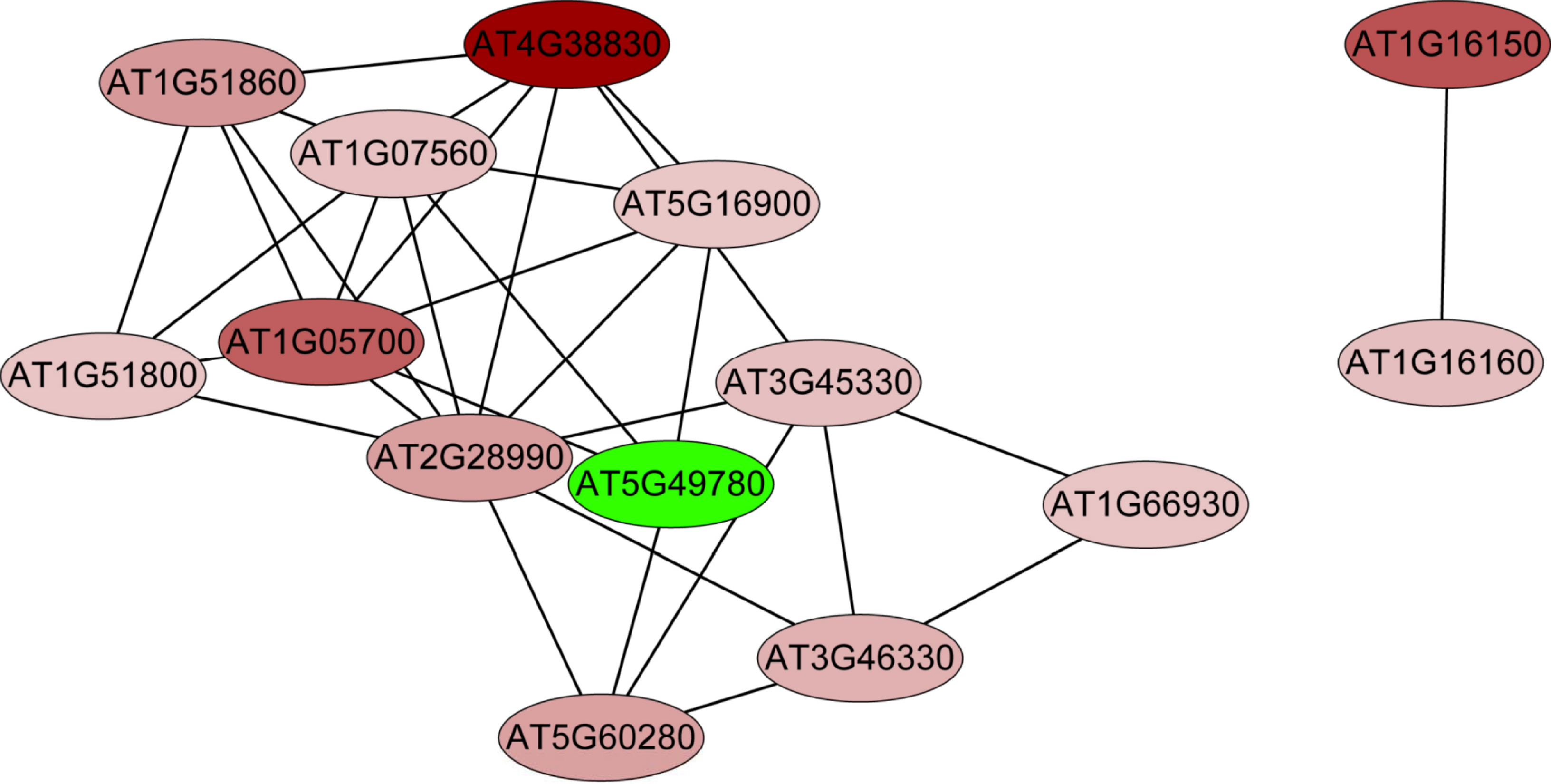

Figure A4-Coexpression relationships of genes with fold change of more than 1.5 fold
