# Supplementary material for: A Digital Compendium of Genes Mediating the Reversible Phosphorylation of Proteins in Fe-Deficient Arabidopsis Roots
Source: Front Plant Sci. 2013 Jun 3;4:173. doi: 10.3389/fpls.2013.00173 (PMC3669753; doi:10.3389/fpls.2013.00173)
Supplement: Table S1 — Differentially expressed protein kinase and phosphatase genes upon iron deficiency. The corresponding response ratios, defined as the transcript level (Reads Per Kilobase per Million mapped reads) in the −Fe treatment divided by the level in the +Fe treatment, are shown in three biological repeats, as well as the mean (P < 0.05). [file 52747_Lan_DataSheet1.ZIP › 52747_Lan_Supplementary_Tables.pdf]

Table A1 Differentially expressed protein kinase and phosphatase genes upon iron deficiency. The corresponding response ratios, defined as the transcript level (Reads Per Kilobase per Million mapped reads) in the –Fe treatment divided by the level in the +Fe treatment, are shown in three biological repeats, as well as the mean (P<0.05).

AGI	Bio1(-fe/+fe)	Bio2(-fe/+fe)	Bio3(-fe/+fe)	Mean(-fe/+fe)	SD
Differentially expressed protein kinase genes upon Fe deficiency					
AT2G19410	10.65	10.94	9.17	10.26	0.95
AT5G53450	5.36	5.33	5.04	5.24	0.18
AT5G01060	4.04	5.38	5.61	5.01	0.85
AT1G51870	5.97	4.34	1.99	4.10	2.00
AT1G77280	4.56	4.04	3.65	4.09	0.46
AT4G38830	3.08	4.05	3.48	3.54	0.49
AT1G16120	3.23	2.91	2.12	2.76	0.57
AT5G39000	3.47	2.48	2.30	2.75	0.63
AT1G16150	2.84	3.03	2.28	2.72	0.39
AT5G23170	2.83	2.16	2.86	2.62	0.40
AT1G05700	2.93	2.71	2.15	2.60	0.40
AT1G51830	2.79	2.42	2.53	2.58	0.19
AT4G26890	2.06	2.32	2.16	2.18	0.13
AT5G07280	2.44	2.04	1.94	2.14	0.27
AT1G33260	2.25	1.92	1.92	2.03	0.19
AT1G51860	2.36	1.96	1.73	2.02	0.32
AT1G72540	2.13	1.59	2.19	1.97	0.33
AT2G28990	1.98	2.02	1.87	1.96	0.08
AT5G60280	2.34	1.79	1.67	1.93	0.36
AT4G18700	1.78	2.01	1.95	1.91	0.12
AT2G30360	1.76	2.13	1.77	1.89	0.21
AT2G45590	1.54	2.17	1.94	1.89	0.32
AT1G51620	1.71	1.28	2.37	1.78	0.55
AT1G08650	1.65	1.96	1.73	1.78	0.16
AT3G46330	2.08	1.52	1.74	1.78	0.28
AT5G35580	2.24	1.54	1.43	1.73	0.44
AT1G01140	1.59	2.13	1.41	1.71	0.37
AT3G49370	1.48	2.05	1.59	1.71	0.30
AT3G27580	1.90	1.48	1.70	1.69	0.21
AT1G16160	1.54	2.02	1.36	1.64	0.34
AT3G45330	1.83	1.45	1.59	1.62	0.19
AT5G55560	1.68	1.92	1.24	1.62	0.34
AT3G57740	1.36	2.07	1.40	1.61	0.39
AT1G07560	1.77	1.67	1.37	1.61	0.21
AT5G25440	1.72	1.41	1.63	1.59	0.16
AT1G51800	1.66	1.63	1.44	1.58	0.12
AT1G66930	1.60	1.71	1.41	1.57	0.15

AT5G35750	1.53	1.55	1.59	1.56	0.03
AT5G16900	1.68	1.60	1.40	1.56	0.15
AT4G04700	1.62	1.68	1.38	1.56	0.16
AT1G74360	1.67	1.69	1.30	1.56	0.22
AT2G46700	1.51	1.63	1.42	1.52	0.11
AT4G11530	1.76	1.30	1.35	1.47	0.25
AT2G35890	1.30	1.31	1.71	1.44	0.23
AT1G16130	1.65	1.37	1.30	1.44	0.19
AT1G69790	1.45	1.54	1.32	1.44	0.11
AT1G51890	1.38	1.46	1.40	1.42	0.04
AT3G27560	1.45	1.29	1.44	1.40	0.09
AT1G25390	1.72	1.28	1.17	1.39	0.29
AT4G18640	1.48	1.32	1.32	1.37	0.10
AT3G59700	1.35	1.37	1.34	1.35	0.02
AT4G14580	1.14	1.60	1.30	1.35	0.23
AT1G51850	1.43	1.31	1.30	1.35	0.08
AT5G35370	1.46	1.39	1.18	1.35	0.14
AT5G20050	1.28	1.46	1.30	1.34	0.10
AT5G61570	1.54	1.29	1.18	1.34	0.19
AT3G22750	1.23	1.34	1.43	1.33	0.10
AT1G45160	1.36	1.35	1.29	1.33	0.04
AT5G45840	1.26	1.47	1.27	1.33	0.12
AT3G04910	1.33	1.37	1.29	1.33	0.04
AT3G07070	1.39	1.21	1.36	1.32	0.10
AT3G21340	1.34	1.29	1.29	1.31	0.03
AT1G51790	1.36	1.32	1.23	1.31	0.07
AT2G28960	1.40	1.31	1.21	1.31	0.09
AT1G27320	1.42	1.26	1.23	1.30	0.10
AT5G51270	1.28	1.30	1.32	1.30	0.02
AT5G57035	1.32	1.29	1.28	1.30	0.02
AT1G72300	1.27	1.29	1.27	1.28	0.01
AT3G28690	1.14	1.29	1.40	1.28	0.13
AT2G43700	1.34	1.31	1.18	1.28	0.09
AT5G62310	1.35	1.15	1.31	1.27	0.10
AT5G06839	1.38	1.30	1.10	1.26	0.14
AT1G07870	1.25	1.37	1.15	1.26	0.11
AT1G30640	1.19	1.26	1.31	1.25	0.06
AT3G50070	1.28	1.14	1.33	1.25	0.10
AT3G45410	1.20	1.17	1.38	1.25	0.11
AT5G65530	1.28	1.32	1.15	1.25	0.08
AT3G17410	1.34	1.29	1.12	1.25	0.12
AT5G41990	1.29	1.20	1.25	1.25	0.05
AT5G44100	1.09	1.27	1.36	1.24	0.14
AT3G45240	1.14	1.21	1.31	1.22	0.09

AT2G19130	1.27	1.09	1.27	1.21	0.11
AT1G64300	1.14	1.33	1.15	1.21	0.11
AT1G56145	1.22	1.21	1.18	1.20	0.02
AT2G39660	1.19	1.22	1.20	1.20	0.02
AT3G05050	1.09	1.24	1.28	1.20	0.10
AT3G46930	1.19	1.37	1.05	1.20	0.16
AT4G18710	1.15	1.18	1.27	1.20	0.06
AT3G55450	1.21	1.13	1.24	1.19	0.06
AT1G63500	1.19	1.18	1.22	1.19	0.02
AT1G56140	1.25	1.16	1.17	1.19	0.05
AT1G70530	1.23	1.14	1.17	1.18	0.05
AT3G21630	1.26	1.14	1.14	1.18	0.07
AT1G63700	1.32	1.13	1.10	1.18	0.12
AT5G03320	1.12	1.20	1.23	1.18	0.06
AT1G03930	1.21	1.20	1.11	1.17	0.06
AT1G10940	1.15	1.18	1.16	1.16	0.02
AT1G61590	1.21	1.18	1.10	1.16	0.05
AT3G61160	1.19	1.23	1.06	1.16	0.09
AT1G03920	1.15	1.06	1.26	1.16	0.10
AT3G07980	1.24	1.02	1.20	1.15	0.12
AT2G46340	1.08	1.12	1.26	1.15	0.10
AT4G33430	1.06	1.22	1.17	1.15	0.08
AT5G03730	1.19	1.09	1.16	1.15	0.05
AT1G09440	1.20	1.10	1.13	1.14	0.05
AT4G08850	1.21	1.12	1.10	1.14	0.06
AT5G15080	1.02	1.24	1.17	1.14	0.11
AT2G02800	1.13	1.13	1.13	1.13	0.00
AT3G13690	1.13	1.14	1.12	1.13	0.01
AT5G44290	1.18	1.05	1.13	1.12	0.06
AT1G79570	1.09	1.09	1.17	1.11	0.05
AT1G18890	1.10	1.17	1.06	1.11	0.05
AT1G53050	1.03	1.13	1.15	1.10	0.06
AT1G09970	1.13	1.13	1.04	1.10	0.05
AT3G20410	1.07	1.14	1.07	1.09	0.04
AT3G14840	1.10	1.10	1.06	1.09	0.02
AT3G25840	1.04	1.08	1.13	1.08	0.05
AT1G18150	1.09	1.08	1.07	1.08	0.01
AT5G63370	1.10	1.05	1.07	1.08	0.03
AT4G02010	0.93	0.99	0.94	0.95	0.03
AT1G69220	0.96	0.92	0.92	0.93	0.03
AT2G42880	0.93	0.93	0.93	0.93	0.00
AT2G37050	0.95	0.89	0.95	0.93	0.03
AT5G51560	0.91	0.92	0.95	0.93	0.02
AT4G26540	0.94	0.90	0.93	0.93	0.02

AT4G23650	0.93	0.93	0.90	0.92	0.02
AT3G51850	0.91	0.94	0.91	0.92	0.01
AT1G02090	0.91	0.91	0.94	0.92	0.02
AT3G19100	0.87	0.96	0.90	0.91	0.05
AT5G56890	0.91	0.91	0.90	0.91	0.01
AT5G10020	0.90	0.88	0.94	0.91	0.03
AT4G22730	0.85	0.90	0.96	0.91	0.05
AT3G18040	0.92	0.91	0.88	0.90	0.02
AT5G55830	0.93	0.85	0.93	0.90	0.05
AT4G08920	0.89	0.86	0.94	0.90	0.04
AT3G58690	0.89	0.95	0.85	0.90	0.05
AT5G63940	0.89	0.85	0.95	0.90	0.05
AT1G09570	0.94	0.87	0.89	0.90	0.04
AT2G41890	0.94	0.90	0.84	0.89	0.05
AT4G22130	0.90	0.84	0.93	0.89	0.05
AT3G49670	0.93	0.87	0.87	0.89	0.03
AT4G36180	0.86	0.89	0.90	0.89	0.02
AT2G44830	0.92	0.84	0.89	0.88	0.04
AT5G18610	0.85	0.91	0.90	0.88	0.03
AT4G28650	0.85	0.89	0.89	0.88	0.02
AT4G23740	0.85	0.83	0.93	0.87	0.06
AT2G35620	0.88	0.83	0.90	0.87	0.03
AT5G14640	0.87	0.85	0.90	0.87	0.03
AT5G21170	0.92	0.83	0.87	0.87	0.05
AT1G68400	0.88	0.88	0.84	0.87	0.02
AT5G58540	0.81	0.92	0.86	0.86	0.06
AT1G18040	0.90	0.94	0.75	0.86	0.10
AT1G30270	0.75	0.93	0.90	0.86	0.10
AT5G16590	0.79	0.86	0.92	0.86	0.07
AT3G23000	0.82	0.87	0.88	0.86	0.03
AT1G52540	0.87	0.80	0.89	0.86	0.05
AT5G63650	0.84	0.81	0.91	0.85	0.05
AT4G16970	0.85	0.85	0.86	0.85	0.01
AT5G18700	0.82	0.84	0.90	0.85	0.04
AT4G20940	0.86	0.79	0.90	0.85	0.06
AT5G08590	0.84	0.82	0.88	0.85	0.03
AT3G19300	0.91	0.82	0.82	0.85	0.05
AT5G59650	0.90	0.72	0.92	0.85	0.11
AT2G01820	0.87	0.79	0.87	0.84	0.04
AT3G06483	0.91	0.75	0.85	0.84	0.08
AT1G20930	0.84	0.82	0.86	0.84	0.02
AT1G16330	0.83	0.84	0.82	0.83	0.01
AT2G16750	0.81	0.86	0.82	0.83	0.03
AT3G06030	0.86	0.79	0.81	0.82	0.04

AT1G07570	0.91	0.72	0.82	0.82	0.10
AT1G21590	0.80	0.79	0.84	0.81	0.03
AT1G61380	0.72	0.88	0.83	0.81	0.08
AT1G35710	0.94	0.68	0.80	0.81	0.13
AT2G01210	0.80	0.78	0.84	0.81	0.03
AT3G48260	0.80	0.74	0.88	0.81	0.07
AT1G34210	0.82	0.77	0.80	0.80	0.02
AT1G10470	0.80	0.73	0.84	0.79	0.05
AT4G01330	0.75	0.75	0.86	0.79	0.07
AT5G59010	0.83	0.77	0.75	0.78	0.04
AT2G26980	0.72	0.75	0.84	0.77	0.06
AT4G14780	0.75	0.76	0.79	0.77	0.02
AT5G21222	0.86	0.69	0.77	0.77	0.08
AT1G75820	0.79	0.78	0.74	0.77	0.03
AT2G26330	0.69	0.71	0.87	0.76	0.10
AT5G10520	0.71	0.77	0.78	0.75	0.04
AT1G73690	0.85	0.61	0.75	0.74	0.12
AT1G16110	0.68	0.73	0.74	0.72	0.03
AT5G16810	0.63	0.85	0.68	0.72	0.11
AT5G06740	0.75	0.74	0.61	0.70	0.08
AT3G45780	0.65	0.69	0.76	0.70	0.06
AT2G48010	0.80	0.71	0.56	0.69	0.12
AT2G46850	0.69	0.63	0.74	0.69	0.06
AT3G50230	0.55	0.65	0.72	0.64	0.09
AT5G49760	0.68	0.62	0.60	0.63	0.04
AT1G61480	0.61	0.67	0.59	0.62	0.04
AT5G49780	0.60	0.65	0.60	0.62	0.03
AT2G25090	0.52	0.70	0.62	0.61	0.09
AT1G07150	0.56	0.76	0.50	0.60	0.13
AT5G59660	0.59	0.57	0.66	0.60	0.05
AT2G18470	0.64	0.55	0.41	0.53	0.11
AT1G74490	0.40	0.42	0.57	0.46	0.09
AT1G21230	0.28	0.50	0.54	0.44	0.14
AT4G40010	0.35	0.45	0.51	0.44	0.08
Differentially expressed protein phosphatase genes upon Fe deficiency					
AT2G01880	3.03	3.57	2.74	3.11	0.42
AT2G32960	1.84	2.17	1.85	1.95	0.19
AT3G49370	1.48	2.05	1.59	1.71	0.30
AT2G01890	1.62	1.54	1.55	1.57	0.04
AT2G46700	1.51	1.63	1.42	1.52	0.11
AT5G01700	1.19	1.28	1.57	1.35	0.20
AT4G03960	1.15	1.24	1.46	1.28	0.16
AT2G28890	1.17	1.30	1.30	1.26	0.08
AT5G10740	1.26	1.21	1.24	1.24	0.02

AT4G27800	1.32	1.13	1.23	1.23	0.10
AT2G03450	1.22	1.22	1.13	1.19	0.05
AT1G05000	1.23	1.08	1.24	1.18	0.09
AT1G34750	1.23	1.18	1.12	1.18	0.05
AT5G24270	1.15	1.30	1.05	1.17	0.12
AT3G51370	1.08	1.21	1.21	1.17	0.08
AT5G27840	1.14	1.28	1.07	1.16	0.11
AT5G23720	1.25	1.11	1.12	1.16	0.08
AT5G53140	1.09	1.12	1.21	1.14	0.07
AT3G09100	1.20	1.03	1.16	1.13	0.09
AT5G11860	1.19	1.09	1.08	1.12	0.06
AT1G47380	1.10	1.10	1.14	1.11	0.03
AT2G35680	1.10	1.07	1.16	1.11	0.05
AT3G10550	1.12	1.11	1.09	1.11	0.02
AT1G07630	1.10	1.15	1.07	1.10	0.04
AT3G19420	1.09	1.04	1.06	1.06	0.03
AT1G09160	0.96	0.89	0.92	0.92	0.03
AT3G19100	0.87	0.96	0.90	0.91	0.05
AT3G58500	0.96	0.87	0.90	0.91	0.04
AT2G42500	0.90	0.86	0.91	0.89	0.03
AT3G50110	0.89	0.84	0.94	0.89	0.05
AT3G09970	0.87	0.84	0.85	0.85	0.02
AT5G50400	0.85	0.76	0.84	0.82	0.05
AT3G16560	0.79	0.83	0.80	0.81	0.02
AT1G16220	0.80	0.81	0.78	0.80	0.02
AT3G52180	0.91	0.70	0.76	0.79	0.11
AT2G30170	0.64	0.82	0.75	0.73	0.09
AT2G16430	0.68	0.68	0.76	0.71	0.05
AT5G26010	0.73	0.60	0.61	0.65	0.07
AT5G59220	0.41	0.49	0.46	0.45	0.04

Table A2 Genes associated with the major module FEPKPP1

AGI	Function	Subfamily
AT1G05700	leucine-rich repeat protein kinase, putative	LRR-I
AT1G07560	leucine-rich repeat protein kinase, putative	LRR-I
AT1G09970	LRR XI-23; ATP binding / kinase/ protein kinase/ protein serine/threonine kinase	LRR-XI
AT1G51790	kinase	LRR-I
AT1G51800	leucine-rich repeat protein kinase, putative	LRR-I
AT1G51830	ATP binding / kinase/ protein serine/threonine kinase	LRR-I
AT1G51850	leucine-rich repeat protein kinase, putative	LRR-I
AT1G51860	leucine-rich repeat protein kinase, putative	LRR-I
AT1G51890	leucine-rich repeat protein kinase, putative	LRR-I
AT1G56140	leucine-rich repeat family protein / protein kinase family protein	LRR-VIII-2
AT1G56145	leucine-rich repeat family protein / protein kinase family protein	LRR-VIII-2
AT1G66930	serine/threonine protein kinase family protein	LRK10L-2
AT1G72300	leucine-rich repeat transmembrane protein kinase, putative	LRR-Xb
AT2G19130	S-locus lectin protein kinase family protein	SD-2b
AT2G28960	leucine-rich repeat protein kinase, putative	LRR-I
AT2G28990	leucine-rich repeat protein kinase, putative	LRR-I
AT2G48010	RKF3 (RECEPTOR-LIKE KINASE IN IN FLOWERS 3); kinase/ receptor signaling protein serine/threonine kinase	RKF3
AT3G14840	leucine-rich repeat family protein / protein kinase family protein	LRR-VIII-2
AT3G21630	CERK1 (CHITIN ELICITOR RECEPTOR KINASE 1); kinase/ receptor signaling protein/ transmembrane receptor protein kinase	LysM-I
AT3G45330	lectin protein kinase family protein	L-LEC
AT3G45410	lectin protein kinase family protein	L-LEC

AT3G46330	MEE39 (maternal effect embryo arrest 39); kinase	LRR-I
AT3G59700	ATHLECRK (ARABIDOPSIS THALIANA LECTIN-RECEPTOR KINASE); kinase	L-LEC
AT4G08850	kinase	LRR-XII
AT4G11530	kinase	DUF26
AT4G18640	MRH1 (morphogenesis of root hair 1); ATP binding / protein binding / protein kinase/ protein serine/threonine kinase/ protein tyrosine kinase	LRR-VI-2
AT4G38830	protein kinase family protein	DUF26
AT5G06740	lectin protein kinase family protein	L-LEC
AT5G16900	protein kinase, putative	LRR-I
AT5G35370	leucine-rich repeat protein kinase, putative	SD-2b
AT5G49780	WNK8 (WITH NO LYSINE (K) KINASE 8); kinase/ protein kinase	LRR-VIII-1
AT5G58540	protein kinase family protein	LRR-VI-2
AT5G60280	protein kinase family protein	L-LEC
AT1G07870	protein kinase family protein	RLCK-VIIa
AT1G61590	protein kinase, putative	RLCK-VIIa
AT2G16750	protein kinase family protein	RLCK-VI
AT2G39660	BIK1 (BOTRYTIS-INDUCED KINASE1); kinase	RLCK-VIIa
AT3G17410	serine/threonine protein kinase, putative	RLCK-VIII
AT3G55450	protein kinase, putative	RLCK-VIIa
AT5G03320	protein kinase, putative	RLCK-VIIa
AT5G10520	bZIP family transcription factor	RLCK-VI
AT5G15080	RBK1 (Rop Binding protein Kinases 1); ATP binding / kinase/ protein kinase/ protein serine/threonine kinase	RLCK-VIIa
AT5G35580	ATP binding / carbohydrate binding / kinase/ protein kinase/ protein serine/threonine kinase/ protein tyrosine kinase/ sugar binding	RLCK-VIIa
AT5G65530	lectin protein kinase family protein	RLCK-VI
AT1G30270	CIPK23 (CBL-INTERACTING PROTEIN KINASE 23); kinase/ protein binding / protein serine/threonine kinase	CAMK_AMPK
AT4G14580	CIPK4 (CBL-interacting protein kinase 4); ATP binding / kinase/ protein kinase/ protein serine/threonine kinase	CAMK_AMPK

**AT3G20410**	CPK9 (calmodulin-domain protein kinase 9); calmodulin-dependent protein kinase/ kinase	CAMK_CDPK
**AT5G41990**	ATP binding / kinase/ protein kinase/ protein serine/threonine kinase/ protein tyrosine kinase	Other_WNK
**AT5G55560**	ATP binding / kinase/ protein serine/threonine kinase	Other_WNK
**AT3G46930**	protein kinase family protein	RK_RAF-like2
**AT1G07630**	PLL5; catalytic/ protein serine/threonine phosphatase	
**AT1G34750**	protein phosphatase 2C, putative / PP2C, putative	
**AT2G03450**	PAP9 (PURPLE ACID PHOSPHATASE 9); acid phosphatase/ protein serine/threonine phosphatase	
**AT2G28890**	PLL4 (POLTERGEIST LIKE 4); catalytic/ protein serine/threonine phosphatase	
**AT2G35680**	dual specificity protein phosphatase family protein	
**AT3G58500**	PP2A-3 (PROTEIN PHOSPHATASE 2A-3); protein serine/threonine phosphatase	
**AT5G24270**	SOS3 (SALT OVERLY SENSITIVE 3); calcium ion binding / calcium-dependent protein serine/threonine phosphatase	

Table A3 Genes associated with the module FEPKPP2

AGI	Function	Mean(-fe/+fe)	SD
**AT3G58810**	ATMTP3, ATMTPA2, MTP3, MTPA2, metal tolerance protein A2	17.09	1.35
**AT4G19370**	Protein of unknown function (DUF1218)	13.36	4.97
**AT3G13610**	2-oxoglutarate (2OG) and Fe(II)-dependent oxygenase superfamily protein	10.33	0.77
**AT5G03570**	ATIREG2, FPN2, IREG2, iron regulated 2	7.74	0.84
**AT4G10510**	Subtilase family protein	7.10	1.59
**AT1G74770**	zinc ion binding	6.35	0.57
**AT2G20030**	RING/U-box superfamily protein	6.01	1.41
**AT3G60330**	AHA7, HA7, H(+)-ATPase 7	4.11	0.72
**AT5G45080**	AtPP2-A6, PP2-A6, phloem protein 2-A6	3.84	0.17
**AT3G18560**	unknown protein	3.59	0.65
**AT1G18910**	zinc ion binding;zinc ion binding	3.31	0.11
**AT5G40590**	Cysteine/Histidine-rich C1 domain family protein	3.18	0.49
**AT3G47040**	Glycosyl hydrolase family protein	3.18	0.49
**AT5G61250**	AtGUS1, GUS1, glucuronidase 1	2.64	0.21
**AT5G07390**	ATRBOHA, RBOHA, respiratory burst oxidase homolog A	2.38	0.45
**AT3G14470**	NB-ARC domain-containing disease resistance protein	2.29	0.26
**AT1G05250**	Peroxidase superfamily protein	2.28	1.15
**AT1G45145**	ATH5, ATTRX5, LIV1, TRX5, thioredoxin H-type 5	1.98	0.23
**AT4G14630**	GLP9, germin-like protein 9	1.98	0.35
**AT2G21020**	pseudogene, major intrinsic protein (MIP) family	1.97	0.40
**AT5G66985**	unknown protein	1.95	0.44
**AT5G62480**	ATGSTU9, GST14, GST14B, GSTU9, glutathione S-transferase tau 9	1.93	0.17
**AT5G04860**	unknown protein	1.88	0.08
**AT1G70690**	HWI1, PDLP5, Receptor-like protein kinase-related family protein	1.85	0.10
**AT4G25090**	Riboflavin synthase-like superfamily protein	1.85	0.14
**AT3G46270**	receptor protein kinase-related	1.84	0.37
**AT1G29020**	Calcium-binding EF-hand family protein	1.79	0.31
**AT3G54420**	ATCHITIV, ATEP3, CHIV, EP3, homolog of carrot EP3-3 chitinase	1.78	0.53
**AT1G44050**	Cysteine/Histidine-rich C1 domain family protein	1.77	0.17
**AT1G71400**	AtRLP12, RLP12, receptor like protein 12	1.73	0.28
**AT2G35000**	RING/U-box superfamily protein	1.69	0.08

**AT4G22590**	Haloacid dehalogenase-like hydrolase (HAD) superfamily protein	1.68	0.25
**AT1G23140**	Calcium-dependent lipid-binding (CaLB domain) family protein	1.68	0.22
**AT4G20110**	BP80-3;1, VSR3;1, VSR7, VACUOLAR SORTING RECEPTOR 7	1.66	0.09
**AT3G47050**	Glycosyl hydrolase family protein	1.64	0.22
**AT4G26470**	Calcium-binding EF-hand family protein	1.63	0.16
**AT5G44920**	Toll-Interleukin-Resistance (TIR) domain family protein	1.59	0.17
**AT2G26410**	Iqd4, IQ-domain 4	1.59	0.21
**AT3G20960**	CYP705A33, cytochrome P450, family 705, subfamily A, polypeptide 33	1.59	0.26
**AT4G36990**	AT-HSFB1, ATHSF4, HSF4, HSFB1, heat shock factor 4	1.57	0.18
**AT5G22630**	ADT5, arogenate dehydratase 5	1.54	0.17
**AT3G25930**	Adenine nucleotide alpha hydrolases-like superfamily protein	1.54	0.16
**AT5G48290**	Heavy metal transport/detoxification superfamily protein	1.52	0.28
**AT4G26060**	Ribosomal protein L18ae family	1.51	0.07
**AT2G30840**	2-oxoglutarate (2OG) and Fe(II)-dependent oxygenase superfamily protein	1.51	0.21
**AT4G11290**	Peroxidase superfamily protein	0.65	0.03
**AT2G17050**	disease resistance protein (TIR-NBS-LRR class)	0.64	0.02
**AT2G38380**	Peroxidase superfamily protein	0.60	0.04
**AT3G45660**	Major facilitator superfamily protein	0.60	0.08
**AT5G40510**	Sucrase/ferredoxin-like family protein	0.59	0.02

Table A4 Genes associated with the module FEPKPP3

AGI	Function	Mean(-fe/+fe)	SD
AT5G55620	unknown protein	de novo synthesis	
AT3G53280	CYP71B5, cytochrome p450 71b5	128.04	58.05
AT5G67370	Protein of unknown function (DUF1230)	9.75	1.52
AT2G42750	DNAJ heat shock N-terminal domain-containing protein	1.94	0.26
AT4G39970	Haloacid dehalogenase-like hydrolase (HAD) superfamily protein	1.65	0.26
AT5G11420	Protein of unknown function, DUF642	0.67	0.00
AT1G54780	TLP18.3, thylakoid lumen 18.3 kDa protein	0.66	0.14
AT1G12250	Pentapeptide repeat-containing protein	0.66	0.19
AT2G20260	PSAE-2, photosystem I subunit E-2	0.65	0.12
AT1G76080	ATCDSP32, CDSP32, chloroplastic drought-induced stress protein of 32 kD	0.65	0.08
AT5G17170	ENH1, rubredoxin family protein	0.65	0.10
AT1G54500	Rubredoxin-like superfamily protein	0.65	0.01
AT5G04140	FD-GOGAT, GLS1, GLU1, GLUS, glutamate synthase 1	0.64	0.07
AT4G28750	PSAE-1, Photosystem I reaction centre subunit IV / PsaE protein	0.64	0.16
AT3G26060	ATPRX Q, Thioredoxin superfamily protein	0.64	0.10
AT1G52230	PSAH-2, PSAH2, PSI-H, photosystem I subunit H2	0.64	0.11
AT1G44575	NPQ4, PSBS, Chlorophyll A-B binding family protein	0.63	0.01
AT1G03130	PSAD-2, photosystem I subunit D-2	0.62	0.07
AT1G64900	CYP89, CYP89A2, cytochrome P450, family 89, subfamily A, polypeptide 2	0.62	0.07
AT3G56010	unknown protein	0.61	0.05
AT5G64040	PSAN, photosystem I reaction center subunit PSI-N,	0.61	0.05

AT1G08380	PSAO, photosystem I subunit O	0.61	0.06
AT1G03630	POR C, PORC, protochlorophyllide oxidoreductase C	0.61	0.07
AT1G64860	RPOD1, SIG1, SIG2, SIGA, SIGB, sigma factor A	0.60	0.01
AT1G30380	PSAK, photosystem I subunit K	0.59	0.07
AT5G58330	lactate/malate dehydrogenase family protein	0.58	0.07
AT2G38230	ATPDX1.1, PDX1.1, pyridoxine biosynthesis 1.1	0.58	0.10
AT5G51010	Rubredoxin-like superfamily protein	0.55	0.07
AT4G24930	thylakoid lumenal 17.9 kDa protein, chloroplast	0.55	0.04
AT1G22430	GroES-like zinc-binding dehydrogenase family protein	0.54	0.14
AT5G13770	Pentatricopeptide repeat (PPR-like) superfamily protein	0.49	0.06
AT5G59400	unknown protein	0.48	0.15
AT1G60160	Potassium transporter family protein	0.48	0.03
AT4G13250	NYC1, NAD(P)-binding Rossmann-fold superfamily protein	0.46	0.01
AT3G25770	AOC2, allene oxide cyclase 2	0.39	0.08
